# Assessing Mongolian gerbil emotional behavior: effects of two shock intensities and response-independent shocks during an extended inhibitory-avoidance task

**DOI:** 10.7717/peerj.4009

**Published:** 2017-11-13

**Authors:** Camilo Hurtado-Parrado, Camilo González-León, Mónica A. Arias-Higuera, Angelo Cardona, Lucia G. Medina, Laura García-Muñoz, Christian Sánchez, Julián Cifuentes, Juan Carlos Forigua, Andrea Ortiz, Cesar A. Acevedo-Triana, Javier L. Rico

**Affiliations:** 1 Animal Behavior Laboratory, Faculty of Psychology, Fundación Universitaria Konrad Lorenz, Bogota, D.C., Colombia; 2 School of Psychology, Universidad Pedagógica y Tecnológica de Colombia, Tunja, Colombia

**Keywords:** Mongolian gerbil, Step-down, Video fear conditioning system, Inhibitory avoidance, Passive avoidance, Foot shock intensities, Meriones unguiculatus, Exploratory behavior, Emotional behavior

## Abstract

Despite step-down inhibitory avoidance procedures that have been widely implemented in rats and mice to study learning and emotion phenomena, performance of other species in these tasks has received less attention. The case of the Mongolian gerbil is of relevance considering the discrepancies in the parameters of the step-down protocols implemented, especially the wide range of foot-shock intensities (i.e., 0.4–4.0 mA), and the lack of information on long-term performance, extinction effects, and behavioral patterning during these tasks. Experiment 1 aimed to (a) characterize gerbils’ acquisition, extinction, and steady-state performance during a multisession (i.e., extended) step-down protocol adapted for implementation in a commercially-available behavioral package (Video Fear Conditioning System—MED Associates Fairfax, VT, USA), and (b) compare gerbils’ performance in this task with two shock intensities – 0.5 vs. 1.0 mA—considered in the low-to-mid range. Results indicated that the 1.0 mA protocol produced more reliable and clear evidence of avoidance learning, extinction, and reacquisition in terms of increments in freezing and on-platform time as well as suppression of platform descent. Experiment 2 aimed to (a) assess whether an alternate protocol consisting of a random delivery of foot shocks could replicate the effects of Experiment 1 and (b) characterize gerbils’ exploratory behavior during the step-down task (jumping, digging, rearing, and probing). Random shocks did not reproduce the effects observed with the first protocol. The data also indicated that a change from random to response-dependent shocks affects (a) the length of each visit to the platform, but not the frequency of platform descends or freezing time, and (b) the patterns of exploratory behavior, namely, suppression of digging and rearing, as well as increments in probing and jumping. Overall, the study demonstrated the feasibility of the extended step-down protocol for studying steady performance, extinction, and reacquisition of avoidance behavior in gerbils, which could be easily implemented in a commercially available system. The observation that 1.0 mA shocks produced a clear and consistent avoidance behavior suggests that implementation of higher intensities is unnecessary for reproducing aversive-conditioning effects in this species. The observed patterning of freezing, platform descents, and exploratory responses produced by the change from random to periodic shocks may relate to the active defensive system of the gerbil. Of special interest is the probing behavior, which could be interpreted as risk assessment and has not been reported in other rodent species exposed to step-down and similar tasks.

## Introduction

An experimental procedure widely implemented for studying learning and emotion phenomena in rodents is the “step down”, also known as a type of passive or inhibitory avoidance procedure (Izquierdo, Furini & Myskiw, 2016). The protocol generally consists of introducing the subject to an experimental chamber that has a slightly elevated platform above a grid floor, followed by the delivery of a single, several, or continuous foot shocks through the grid floor when the subject descends from the platform. Subjects exposed to this task typically show increments in time spent on the platform and latency to descend to the grid floor (i.e., step-down responses), and suppression of step-down responses.

While step-down procedures have been extensively implemented in rats and mice, the performance of other species in this task has received less attention. The case of the Mongolian gerbil is of particular relevance considering the ample heterogeneity in the methodologies implemented, including dimensions, arrangement, and materials of the apparatuses, and intensities and durations of the foot shocks (e.g., Amano et al., 1993; Galvani, Riddel & Foster, 1975; Karasawa et al., 1990; Ko et al., 2009; Park et al., 2011; Sim et al., 2005; Sudo et al., 1997; Wen et al., 1995).

The extensive discrepancy in the parameters of the aversive stimulus implemented during step-down procedures with Mongolian gerbils (foot shocks) is especially striking. A survey of several studies has shown that the durations of the shocks have ranged from 0.1 s to 3.0 s, and their intensities have varied between 0.4 mA and 4.0 mA (Amano et al., 1993; Galvani, Riddel & Foster, 1975; Galvani & Twitty, 1976; Himi, Ishizaki & Murota, 1998; Karasawa et al., 1990; Ko et al., 2009; Kudo et al., 1992; Matsuda et al., 1997; Pratt et al., 1994; Sim et al., 2005). Such diverseness in the parameters of the foot shocks is alarming in terms of its implications for the validity and commensurability of the associated findings (Lewejohann et al., 2006) and compliance with principles endorsed by agencies that regulate animal experimentation—i.e., efforts to refine procedures to minimize pain and distress (National Research Council, 2011).

### Overview of the study

We designed a step-down protocol for gerbils based on factors reported to have promoted the behavioral adjustment of this species to this task (e.g., shock frequency, platform size, habituation to the experimental chamber; Galvani, 1974; Galvani, Riddel & Foster, 1975; Lippman, Galosy & Thompson, 1970; Ögren & Stiedl, 2015; Osborne, Caul & Vanstrum, 1976; Twitty & Galvani, 1977; Walters & Abel, 1971). Though the most frequently implemented version of this procedure has entailed a single-trial training session and one testing session, additional trials/sessions of different length can also be scheduled during which the subject may be allowed to freely step down and return to the platform (Izquierdo, Furini & Myskiw, 2016). Considering the lack of information on gerbils’ long-term performance during step-down tasks, and the effects of extinction on that performance, our protocol entailed several sessions—hence the term “extended.” We adapted this protocol for implementation in the Video Fear Conditioning System (VFC; MED Associates Inc, 2009), which is a standard instrument that offers automatic scoring of rodent activity and freezing during aversive conditioning procedures (Anagnostaras et al., 2010).

In Experiment 1, the adapted protocol was used to compare the effects of implementing two foot-shock intensities considered in the low-to-mid range (0.5 and 1.0 mA) on gerbils’ (a) rate of step-down responses (platform descents) per session, (b) time spent on the platform per session, and (c) steady-state performance and extinction effects during the step-down task.

The decision to compare these two foot-shock intensities (0.5 vs. 1.0  mA) resulted from consideration of the following features: (a) Ballard, Sänger & Higgins (2001) used an analogous system to the VFC and could only reproduce defensive reactions in gerbils (flinch, vocalize, and jump—Hurtado-Parrado et al., 2015) when the intensity of the shocks was equal to or greater than 1.0 mA; (b) preliminary studies conducted in our laboratory entailing 1.0 mA shocks resulted in reliable reproduction not only of the same defensive responses reported by Ballard, Sänger & Higgins (2001) but also other responses such as running, thigmotaxis, and 360° jumps; and (c) approval of the step-down protocol by our Institutional Animal Care and Use Committee (IACUC) required the explicit effort to test a refined version of the proposed experimental procedure so the stress and pain of the animals could be reduced without compromising the experimental findings; in this case, reliably reproducing avoidance phenomena in gerbils.

Experiment 2 assessed the reliability and versatility of our step-down protocol by replicating and extending the findings of Experiment 1. Its purpose was twofold: first, testing whether an alternate procedure that entailed exposure to random delivery of foot shocks through the grid floor (i.e., response-independent shocks) was sufficient to reproduce the behavioral effects observed in Experiment 1. Second, the fact that freezing responses only accounted for 40% or less of the total session time during Experiment 1 led to the question of which other type of responses the animals were displaying during the task. Accordingly, we characterized gerbils’ exploratory behavior (rearing, digging, jumping, and probing) during the step-down protocol, which has been reported to differ importantly from that of other rodent models when exposed to aversive tasks (e.g., rats and mice; Crawford et al., 1981; Galvani, Riddel & Foster, 1975; Lippman, Galosy & Thompson, 1970; Osborne, Caul & Vanstrum, 1976; Rico et al., 2016).

## Experiment 1: Adaptation of the Step-Down Protocol and Comparison of Two Shock Intensities

### Method

#### Subjects

Four seven-month-old (75–90 g) experimentally naïve male Mongolian gerbils (*M. unguiculatus*) obtained from the Instituto Nacional de Salud (National Institute of Health, Bogota, Colombia) were used in this study. Animals were housed in pairs in polycarbonate cages (42 × 20 × 20 cm), which contained dust-free wood shaving bedding, and were kept in an animal room under a 12 h light/dark cycle (lights on at 6:00 a.m.) with water and standard rodent pellets available ad libitum. The room temperature was maintained at 23 °C with 55% relative humidity. All experimental procedures were performed in accordance with the US National Institute of Health Guide for the Care and Use of Laboratory Animals and were approved by the Institutional Animal Care and Use Committee (IACUC) at Fundación Universitaria Konrad Lorenz (SAC-011-01-2015).

#### Apparatus

##### Video fear conditioning system—VFC (MED Associates Inc, 2009).

**Figure 1:**
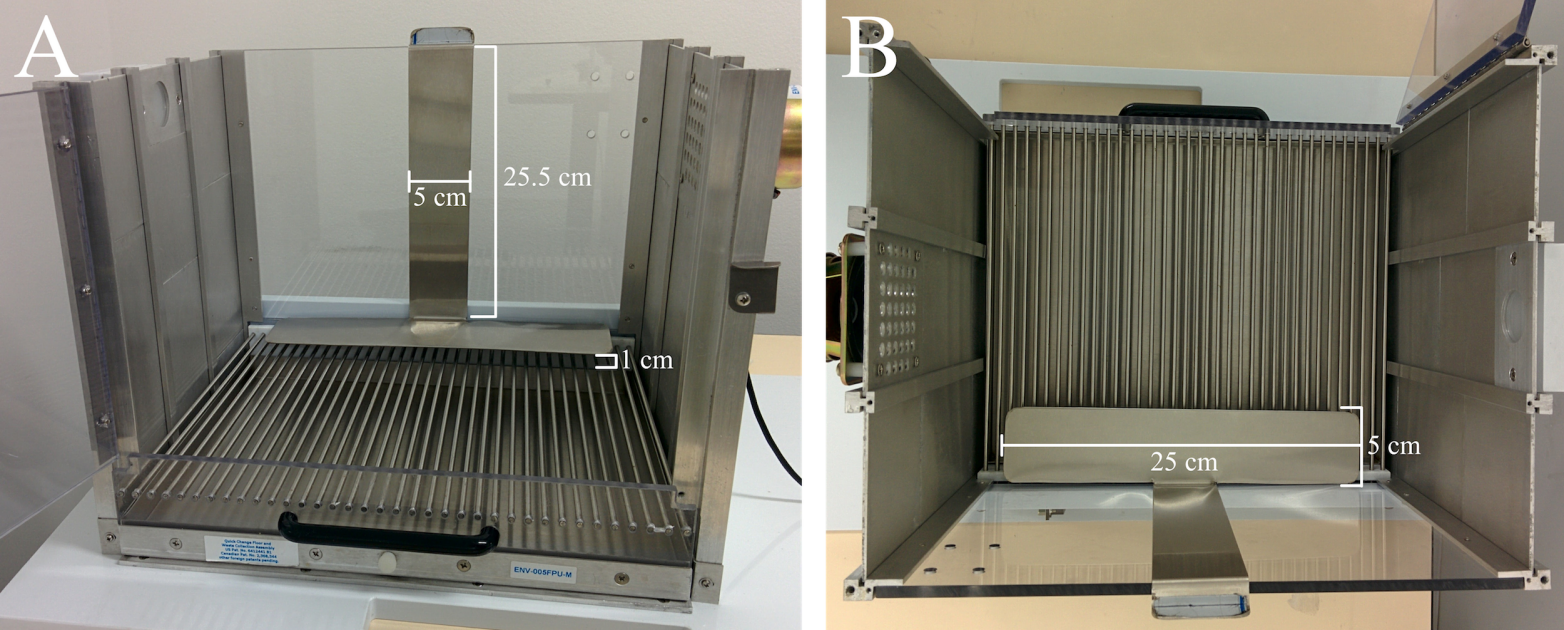
Experimental chamber. Front (A) and top (B) views of the experimental chamber, including location and dimensions of the custom-made platform (25 cm × 5 cm × 0.3 cm) that was hung 1 cm above the grid floor and was secured to the back of the experimental chamber with a wide bar and clip of 5 cm.

A VFC was adapted for the purposes of the present study. A clear polycarbonate (top and front), white acrylic (back), and stainless steel (sides) experimental chamber (32 cm wide, 25 cm high, 25 cm deep; Med Associates Part Number VFC-008) was encased in a white sound-attenuating box (63.5 cm wide, 35.5 cm high, 76 cm deep; Med Associates Part Number NIR-022MD). Inside the experimental chamber were located a stainless-steel grid floor (36 rods, each rod 2 mm diameter, 8 mm center to center; Med Associates Part Number ENV-005FPU-M) and drop-pan. A custom-made stainless-steel platform (25 cm × 5 cm × 0.3 cm) was hung 1 cm above the grid floor, which was secured to the back of the experimental chamber with a bar and clip 5 cm wide (see Fig. 1).

The interior of the experimental chamber was illuminated by overhead near-infrared light of 940 nm (Med Associates NIR-100). Background noise (65 dBA) was provided by a ventilation fan. Video images of the behavioral sessions were recorded at a frame rate of 30 frames per second (640 × 480 pixels) with a video camera equipped with a visible light filter (VID- CAM-MONO-2A). Appendix S1 shows a sample video frame.

A general activity index (Motion Index) was derived in real time from the video stream using computer software (Video Freeze^®^; Med Associates Part Number SOF-843). The Motion Index was subjected to a threshold to generate automatic freezing scores (frequency, duration, and percentage of session time). The freezing threshold was set to the default Minimum Freeze Duration setting of the VFC system (1 s = 30 frames). Though this value was originally validated by Anagnostaras et al. (2010) with mice, preliminary analyses in our laboratory, analogous to those conducted by Anagnostaras et al. (2010), support the feasibility of implementing the same criterion with Mongolian gerbils.

An aversive stimulator/scrambler (Med Associates Part Number ENV-414S) connected to the grid floor harness delivered 0.5 s foot shocks of two intensities (0.5 or 1.0 mA; see *Design* and *Procedure* sections for details).

##### JWatcher+ Video 1.0 (Blumstein & Daniel, 2007).

Using the video files produced by the VFC for each experimental session, step-up and step-down responses and the time spent by each subject on and off the platform were manually scored and analyzed using the JWatcher+Video 1.0, which is a freeware available at http://www.jwatcher.ucla.edu (Blumstein & Daniel, 2007; University of California & Macquarie University). Necessary files for scoring the videos obtained from the VFC using the JWatcher (global and focal behavioral definition files) are available as Appendix S2.

#### Design

A single-case experimental design was implemented in Experiment 1 (Johnston & Pennypacker, 2009; Perone & Hursh, 2013). The decision to use this approach resulted from consideration of the advantages offered by this design for the purposes of the first experiment compared to a group design (Hurtado-Parrado & Lopez-Lopez, 2015), namely, (a) requiring a reduced number of subjects, which aligned with the recommendations of IACUC, (b) studying in depth—i.e., with repeated measures and until subjects reached stable performance—the acquisition and extinction of the behavioral phenomena of interest under the two variations of the chosen parameter (foot shock intensity, namely 0.5 and 1.0 mA), and (c) establishing feasible steady-state performance criteria extrapolated from a wide range of behavioral research conducted with this type of design using animal models (Perone & Hursh, 2013; see Stability criteria below).

The experiment consisted of four baseline (BL1, BL2, BL3, and BL4) and two treatment conditions (*0.5 mA* and *1.0 mA*). During the baseline sessions, no foot shocks were scheduled, and during the treatment sessions, one of the two intensities of the shocks was scheduled. The experiment was initiated with BL1, which provided data on frequency and duration of the gerbil’s visits to the platform, prior to any foot-shock exposure, and served as a habituation period to the apparatus. Three additional baselines (BL2–BL4) were interspersed between treatment conditions, which allowed to identify extinction effects.

Two treatment conditions were scheduled for each shock intensity. Subject 1 and 2 differed from Subject 3 and 4 regarding the sequence to which they were exposed to the treatment conditions. For example, S1 was first exposed to two treatment conditions in which the foot shocks were 0.5 mA, prior to experiencing the two 1.0 mA conditions (see Appendix S3 for the specific sequence of each subject).

Since one aim of the study was to establish the effect of each shock intensity (0.5 versus 1.0 mA) on the steady-state performance of gerbils during the task, baseline and treatment conditions were continued until stability criteria were reached or a maximum number of daily sessions per baseline or treatment conditions was completed.

#### Stability criteria

An experimental condition of the study (baseline or treatment) was concluded for each subject when stability for the total time on the platform per session was reached. Stability criteria were based on those implemented by Critchfield et al. (2003), Green & Estle (2003), and Magoon & Critchfield (2008). The last four sessions of an ongoing phase were daily assessed for each subject to establish if its time allocation to the platform was stable. These four sessions were divided in two blocks of two sessions each (first and last), and means for each block and for the four sessions were calculated. Steady performance was assumed when (a) the difference in means between the first and last block of sessions differed by no more than 15% of the four-session mean (i.e., any change in level between the first and the last block of sessions was within 15% of the four-session average), and (b) no clear trend during the last four sessions was evident by visual inspection of the graphed data (e.g., ascending or descending). An experimental condition was concluded if 15 sessions were conducted, independently of the stability criteria being reached.

#### Procedure

Daily experimental sessions were conducted consecutively between 10:00 and 16:00 h (part of the light cycle of the animals). The total number of sessions scheduled for each subject depended on the application of the stability criteria (see above), and ranged between 76 to 79. The length of each session was 630 s, and only one animal at a time was exposed to the procedure. The experimental chamber, including the grid floor and platform, were cleaned at the end of each subject’s session with a 10% ethanol solution and then dried with a cloth. A description of each experimental condition is provided as follows.

##### Baseline (BL).

Each session of a baseline condition initiated immediately after the subject was introduced to the experimental chamber of the VFC and was placed on the platform. During the 630 s of session time, no shocks were presented. Immediately after the session was terminated, the animal was removed from the experimental chamber and returned to the housing cage. In addition to the automatic measures of immobility provided by the VFC (total duration and frequency per session in seconds), the amount of time that the animal spent on the platform during each visit (in seconds), percentage of total session time spent on the platform, and number of step-down responses were manually scored using the JWatcher+Video 1.0 software (average 95% interobserver agreement was established; Johnston & Pennypacker, 1993). Baseline sessions were continued until the number of step-down responses and total time on the platform reached stability (see Stability criteria above).

##### Foot shocks of 0.5 mA.

Each session was initiated when the subject was introduced to the experimental chamber and placed on the platform. Starting on second 30 (habituation period), foot shocks with an intensity of 0.5 mA and a duration of 0.5 s were delivered every 3 s (shock-shock interval = 3 s). Immediately after the session was terminated, the animal was removed from the experimental chamber and returned to the housing cage. The VFC file necessary for implementing this protocol is available as Appendix S2. The same measurements described for baseline conditions were conducted (see above). This condition was continued until the time on the platform reached stability across sessions or 15 sessions were conducted (see Stability criteria section above).

##### Foot shocks of 1.0 mA.

The same procedure described for each session of the 0.5 mA condition was conducted, with the only difference being a foot-shock intensity of 1.0 mA.

#### Data analyses

We established the magnitude and statistical significance of the effect of each shock intensity (0.5 and 1.0 mA) per subject using *Tau-U* effect-size indices (Parker et al., 2011); to that aim, we used the tool developed by Vannest et al. (2016) (Available at http://www.singlecaseresearch.org/calculators/tau-u). Tau-U index is a method developed for single-case experimental designs, which is based on Kendall’s Rank Correlation and the Mann–Whitney *U* tests. A Tau-U score is interpreted as the percentage of data nonoverlap between baseline and treatment phases. Tau-U was chosen because it addresses trends in baseline phases and has shown equal or higher discriminability and sensitivity than other indices (e.g., PND and IRD—Rakap, 2015). Tau-U scores can be interpreted using the following criteria: .65 or lower = weak effect; .66 to .92 = medium to high effect; and above .93 = large effect.

As it is standard in single-case research (Bourret & Pietras, 2013; Hurtado-Parrado & Lopez-Lopez, 2015), visual analyses of graphed data for each individual were conducted using guidelines for the type of experimental design implemented (e.g., differences in level and variations in trend across baseline and treatment conditions; Bourret & Pietras, 2013). These analyses aimed to provide an in-depth assessment of the relationships between the two shock intensities (0.5 and 1.0 mA) and the three measures of interest (percentage of total session time on platform and freezing, and rate of step-down).

### Results

Table 1 shows effect-size scores (Tau-U) and corresponding statistical significance (*p* values) for each shock intensity (0.5 mA and 1.0 mA) per subject and for each measure of interest—i.e., % of session time on platform, % of session time freezing, and step-down rate.

**Table 1:** Effect-size indices for each shock intensity. Effect-size indices (Tau-U) for each shock intensity (0.5 mA and 1.0 mA) per subject and for each measure of interest (% of session time on platform, % of session time freezing, and step-down rate).

			Subject			
			S1	S2	S3	S4
% session time on platform	0.5 mA	Tau-U	.8290^#^	1.1497^*^	1.1846^*^	.7194^#^
		*p*	<.0001	<.0001	<.0001	.0002
	1.0 mA	Tau-U	1.2779^*^	1.1649^*^	1.2324^*^	.8114^#^
		*p*	<.0001	<.0001	<.0001	.0002
% session time freezing	0.5 mA	Tau-U	.8905^#^	.4686^+^	.8377^#^	.5686^+^
		*p*	<.0001	.0346	<.0001	.0035
	1.0 mA	Tau-U	.8928^#^	1.1206^*^	1.3031^*^	0.8411^#^
		*p*	<.0001	<.0001	<.0001	.0001
Step-down rate	0.5 mA	Tau-U	.0662^+^	.7417^#^	.8838^#^	.2041^+^
		*p*	.7390	.0008	<.0001	.2938
	1.0 mA	Tau-U	.7013^#^	.7949^#^	.8957^#^	.9630^*^
		*p*	.0005	.0001	<.0001	<.0001

**Notes.**

Note: Tau-U scores for each intensity (0.5 mA and 1.0 mA) correspond to weighted averages of the two corresponding treatment conditions (i.e., omnibus effect size). See ‘Design’ in ‘Method’ section.

+ low effect.

# medium-high effect.

* large effect.

**Figure 2:**
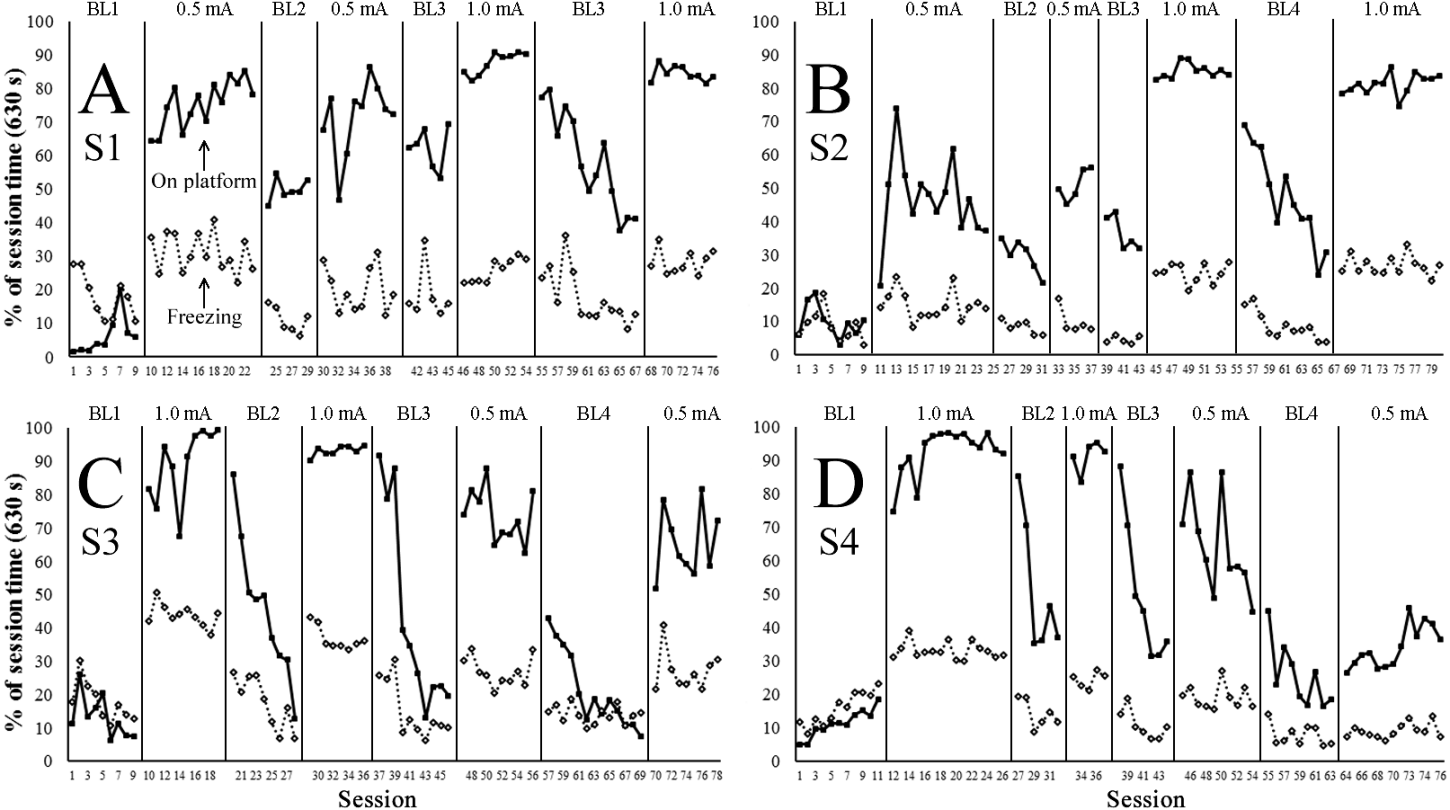
Percentage of the session time spent on the platform and freezing across the baseline and treatment conditions. (A–D) shows for each subject (S1–S4) the percentage of the session time (630 s = 100%) spent on the platform (solid line) and freezing (dashed line) across the baseline (BL1–BL4) and treatment conditions (0.5 mA or 1.0 mA). Note: S1 and S2 differed from S3 and S4 regarding the order in which they were exposed to the treatment conditions— i.e., S1 and S2 initiated the experiment with foot shocks of 0.5 mA, whereas S3 and S4 received 1.0 mA shocks first.

**Figure 3:**
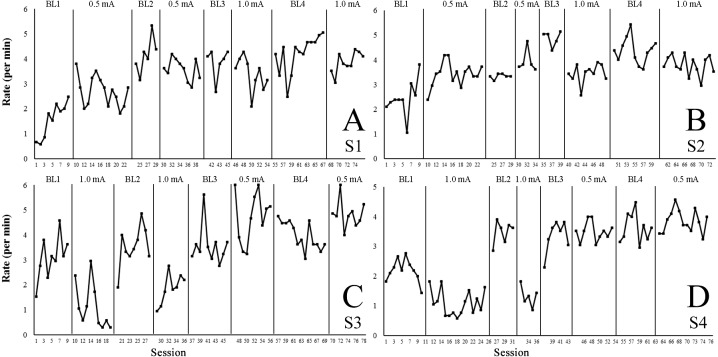
Rate of step-down responses per minute across the baseline and treatment conditions. (A–D) shows for each subject (S1–S4) the rate of step-down responses (platform descents) per minute across the baseline (BL1–BL4) and treatment conditions (0.5 mA or 1.0 mA). Note: S1 and S2 differed from S3 and S4 regarding the order in which they were exposed to the treatment conditions—i.e., S1 and S2 initiated the experiment with foot shocks of 0.5 mA, whereas S3 and S4 received 1.0 mA shocks first.

Figures 2A–2D shows the percentage of session time (630 s = 100%) spent by each subject (S1–S4) on the platform (solid line) and freezing (dashed line) during each session of baseline (panels BL1, BL2, BL3, and BL4) and treatment conditions (0.5 mA and 1.0 mA). Figures 3A–3D show the rate of step-down responses per minute for each subject across the baseline and treatment conditions. Note that S1 and S2 differed from S3 and S4 regarding the order in which they were exposed to the treatment conditions—i.e., S1 and S2 initiated the experiment with foot shocks of 0.5 mA, whereas S3 and S4 received 1.0 mA shocks first.

#### On-platform time

Effect-size data on Table 1 show that both shock intensities overall produced statistically significant medium-to-large effects in time allocations to the platform. However, scores associated to 1.0 mA were consistently stronger across subjects.

A phase-by-phase visual analysis of data on Fig. 2 showed that by the end of the first baseline—i.e., before any exposure to the foot shocks—all subjects were spending less than 20% of the session time on the platform (BL1). Time allocations to the platform increased with the introduction of the first treatment condition (0.5 mA for S1 and S2 and 1.0 mA for S3 and S4). Though this effect was observed in all subjects, it was more salient in subjects exposed to the1.0 mA condition (S3 and S4), which by the end of that phase were allocating more than 90% of the session time to the platform area.

Introduction of the second baseline (BL2) allowed us to observe the effects of extinction on subjects’ time allocation to the platform—i.e., shocks were no longer delivered through the grid floor. Though this manipulation produced decrements in the time all subjects spent on the platform across sessions, only S3’s time allocations to the platform reached BL1 levels.

Replication of the first treatment condition allowed to test reacquisition of the step-down task with the same shock intensity. This manipulation produced an immediate reestablishment of on-platform times at similar levels observed during the initial treatment phase. Likewise, introduction of a second extinction phase (BL3) produced an important reduction in on-platform time that was similar to that observed during the first extinction phase (BL2).

The third and fourth treatment conditions for all subjects consisted of an exposure to the opposite intensity of the shocks. In the case of S1 and S2, which switched to higher-intensity shocks (from 0.5 mA to 1.0 mA), on-platform times increased above previously observed levels and gained stability across the sessions. The opposite pattern was observed in S3 and S4; namely, on-platform times fluctuated markedly across 0.5 mA sessions and were clearly below those observed for the previous treatment conditions with 1.0 mA.

#### Freezing

As shown in Table 1, Tau-*U* scores for 0.5 mA conditions ranged between .47 and .89 across subjects, indicating low to high effects of this manipulation. Tau-*U* scores for the 1.0 mA treatment were consistently higher; they ranged between .84 and 1.30, indicating medium to large effects.

Freezing data shown in Fig. 2 (dashed lines) shows that, previous to any exposure to the foot shocks (BL1), subjects’ immobility accounted for less than a third part of the total session time. Though freezing of all subjects increased above BL1 levels with the introduction of the first treatment condition, this effect was more pronounced in subjects initially exposed to 1.0 mA shocks (i.e., S3 and S4).

All subjects showed a progressive decrement in immobility throughout the first extinction condition (i.e., BL2), which reached levels characteristic of the last sessions of BL1. Though reinstatement of shocks during the replication of the first treatment condition produced in all animals an initial increase in freezing, the immobility of S1 and S2 did not clearly differ from that observed at the end of previous baselines. Additionally, the freezing times of these two subjects remained very similar or decreased during the subsequent extinction phase (BL3).

The change from 0.5 mA to 1.0 mA shocks in S1 and S2 overall produced higher and steadier levels of immobility, as compared to levels observed during earlier baselines, 0.5 mA conditions, and the extinction condition scheduled between the two 1.0 mA conditions (i.e., BL4).

The change from 1.0 mA to 0.5 mA shocks produced a somewhat opposite pattern in S3 and S4; namely, lower and more variable levels of freezing were overall observed throughout the 0.5 mA sessions in comparison to the 1.0 mA sessions.

#### Step-down responses

As shown in Table 1, Tau-*U* scores for the 0.5 mA shock intensity varied importantly—.07 to .88 range. In the only two cases in which they were statistically significant (S2 and S3), they indicated low to medium-high effects. Conversely, effect size scores for the 1.0 mA condition were in all cases significant, ranging between .70 and .96—i.e., medium to large effects.

Figure 3 shows that the rate of platform descents of S1 and S2 during both 0.5 mA conditions exhibited marked variability across sessions and did not differ greatly from the BL1 levels. The step-down rates of these subjects did not decrease in variability with the subsequent introduction of the 1.0 mA conditions, and no major differences were noted overall when comparing levels observed during the 0.5 mA and 1.0 mA conditions. The three extinction phases scheduled between these treatment conditions (BL2, BL3, and BL4) rarely produced clear and steady increments in step-down rates above treatment levels.

The step-down rates of S3 and S4 during both 1.0 mA conditions, though fluctuated importantly across the sessions, clearly remained below levels observed during BL1 and subsequent extinction conditions—i.e., BL2 and BL3. Conversely, when shock intensity switched from 1.0 mA to 0.5 mA, step-down rates of these subjects never reached the low levels characteristic of the 1.0 mA conditions. Moreover, these rates did not differ from those observed during the extinction conditions scheduled before and between 0.5 mA treatment conditions (BL3 and BL4).

### Discussion

The extended step-down protocol for gerbils—via implementation in the VFC system—produced clear and reliable evidence of avoidance learning in terms of rapid and major increments in freezing and on-platform time, and decrements in rates of step-down. However, a comparison of the effect sizes of the two shock intensities implemented (0.5 and 1.0 mA) indicated that 1.0 mA shocks resulted in higher and steadier on-platform and freezing times, as well as more pronounced suppression of step-down responses. These findings confirm previous reports that intensities below 1.0 mA do not produce reliable and clear effects in aversive conditioning in gerbils (Ballard, Sänger & Higgins, 2001). They also suggest that implementation of dramatically higher intensities (e.g., 2–4 mA; Amano et al., 1993; Karasawa et al., 1990) is not necessary for reproducing learning and emotional phenomena in gerbils, including immobility and avoidance responses during step-down tasks.

The arrangement of extensive numbers of treatment sessions and foot-shock discontinuance phases permitted the observation of gerbils’ steady step-down performance and its extinction and reacquisition. The on-platform time and freezing measures typically reached stability in fewer sessions during the 1.0 mA conditions than during the 0.5 mA conditions. In the case of platform descents, they typically fluctuated across sessions, even during the 1.0 mA conditions, and very often failed to reach stability by the end of a given condition.

Extinction phases generally produced noticeable effects when such manipulation followed 1.0 mA treatment conditions. The typical course of immobility and on-platform time following cessation of the 1.0 mA shocks consisted of gradual decrements across sessions that in some cases reached baseline levels. In the case of step-down responses, foot-shock cessation produced increments in this response that tended to be more sudden than gradual. Reinstatement of 1.0 mA shocks after extinction periods typically produced rapid reestablishment of freezing, time on the platform, and step-down behavior to levels observed during previous treatment conditions. The reliability of these extinction and reacquisition effects showed promise for further refinement and implementation of the step-down protocol in pharmacological and behavioral neuroscience research of emotional-memory phenomena (e.g., Izquierdo, Furini & Myskiw, 2016; Knox, 2016).

## Experiment 2: Effects of Response-Independent Foot Shocks

Adjustment of the gerbils to the 1.0 mA treatment conditions of Experiment 1 was characterized by substantial time allocations to the platform. However, their freezing responses typically only accounted for 40% or less of the total session time. These data led to the question of which other type of responses the animals were displaying during the task. Accordingly, Experiment 2 aimed to characterize the activity of the subjects during the 1.0 mA step-down protocol, which allowed an evaluation of other behavioral measures related to exploratory behavior (rearing, digging, jumping, and probing). The second aim of Experiment 2 consisted of an additional effort to refine the step-down protocol, in consonance with animal-welfare concerns. We tested whether a protocol that entailed longer sessions (twice the length of the Experiment 1 sessions—20 min) but substantially fewer scheduled foot shocks could produce similar effects to those observed during Experiment 1. Specifically, we established whether occasional response-independent delivery of 1.0 mA foot shocks—using a random-time schedule of 30 s (RT 30 s)—could produce significant increments in on-platform time allocations and freezing periods, and important reductions in step-down rates.

### Method

#### Subjects

Five six-month-old (75–80 g) *experimentally naïve* male Mongolian gerbils (*M. unguiculatus*) obtained from the Instituto Nacional de Salud (Bogota, Colombia) were used in this experiment (different from Experiment 1). Housing, feeding, temperature, and lighting conditions were the same as described for Experiment 1. All experimental procedures were performed in accordance with the US National Institute of Health Guide for the Care and Use of Laboratory Animals and were approved by the Institutional Animal Care and Use Committee (IACUC) at Fundación Universitaria Konrad Lorenz (SAC-018-01-2016).

#### Apparatus

Same described for Experiment 1.

#### Procedure

Daily experimental sessions were conducted between 10:00 and 16:00 h (part of the light cycle of the animals) over the course of six consecutive days. The experimental chamber, including the grid floor and platform, were cleaned at the end of each subject’s session with a 10% ethanol solution and then dried with a cloth.

The first two sessions—10 min each—were aimed at habituating the subjects to manipulation and the experimental chamber. Each animal remained in the experimental chamber for a period of 10 min. No data were collected during these sessions. During the following four days, two treatment conditions were scheduled consisting of two days each. Table 2 presents the sequence of the treatment conditions, the session duration in minutes, and the corresponding notation.

**Table 2:** Sequence of the treatment conditions of Experiment 2.

Condition	Day	Session time	Notation
Foot shocks on RT 30-s schedule	1 and 2	20 min	D1-R; D2-R
Foot shocks every 3 s	3 and 4	10 min	D3-3s; D4-3s

**Notes.**

*RT 30-s* random-time 30-s schedule of foot shock delivery

##### Foot shocks delivered on a random-time schedule of 30 s (RT 30 s).

During each of two 20 min daily sessions (D1-R and D2-R), each subject was introduced to the experimental chamber of the VFC and placed on the platform. Foot shocks with an intensity of 1.0 mA and a duration of 0.5 s were delivered through the grid floor on a schedule of RT 30 s. An RT schedule arranges a constant probability of response-independent events (e.g., light, sound, food, shocks) at the end of recycling constant time periods—e.g., in a RT 10-s schedule of shock delivery, at every second there is a .1 probability of a shock being presented (Bancroft & Bourret, 2008; Catania, 1991). The specific random values of the intervals between foot shocks were produced using the Microsoft EXCEL^®^ macro reported by Bancroft & Bourret (2008). The EXCEL and VFC files necessary for running this protocol are available as Appendix S2. The animal did not receive any foot shocks during the time that it remained on the platform.

##### Foot shocks of 1.0 mA.

The same procedure described in Experiment 1 was conducted during each of the 10 min sessions scheduled for days 3 and 4 of the study (D3-3s and D4-3s), i.e., foot shocks with an intensity of 1.0 mA and a duration of 0.5 s were delivered every 3 s through the grid floor.

#### Behavioral measurements

The same procedure implemented in Experiment 1 for obtaining measures of on-platform time, platform descents, and freezing was employed in Experiment 2. In addition, the following behaviors were scored using the same protocol: (a) average time of each visit to the platform (in seconds), (b) percentage of session time allocated to rearing in the grid floor, (c) rate of digging on the grid floor (per minute), (d) rate of jumping on the grid floor (per minute) and (e) rate of probing from the platform (per minute). Appendix S4 presents the detailed definitions of these behaviors, which were based on Hurtado-Parrado et al. (2015).

#### Data analyses

Repeated-measures ANOVA was implemented to analyze data regarding the percentage of session time on the platform, freezing, and rearing, rates of step down, digging, and jumping, and average duration of visits to the platform. Due to data distribution, rates of probing were analyzed using the Friedman test for ranks. Whenever necessary, Tukey or Student-Newman-Keuls post-hoc tests were used; in those cases, Cohen-d’s effect-size calculations were conducted.

### Results

**Figure 4:**
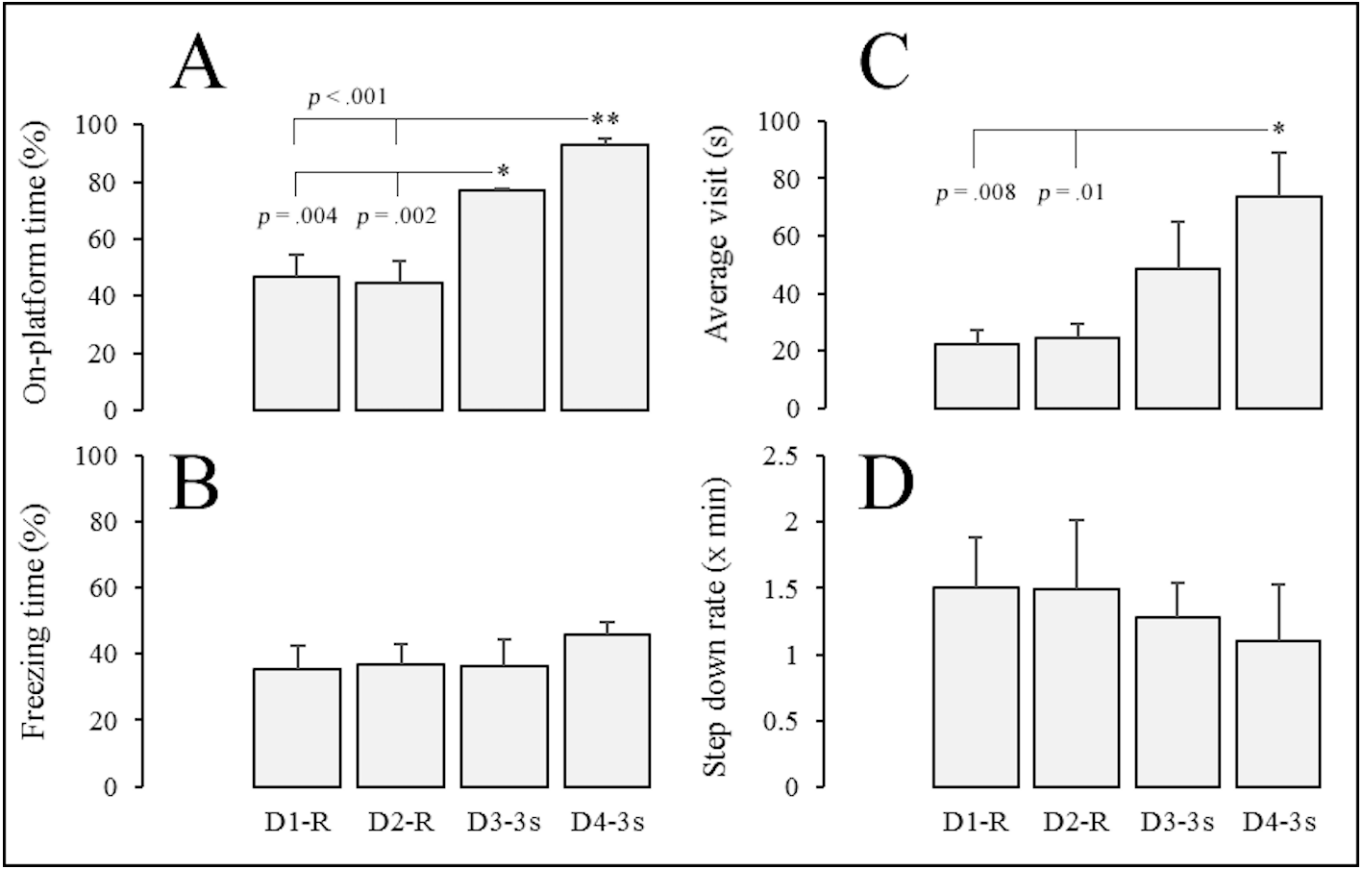
Average percentage of session time spent on the platform and freezing, average duration of visits to the platform in seconds, and rate of step-down responses per minute. Bars in (A) and (B) represent average percentage of session time that gerbils (*n* = 5) spent on the platform and freezing, respectively. (C) shows average duration of visits to the platform in seconds, and (D) the rate of step-down responses per minute. Error bars represent 1 S.E.M. Note: *D*, day of the experiment; *R*, foot shocks delivered with a random-time schedule 30 s (RT 30s); *3 s*, foot shocks delivered every 3 s. ∗, statistically significant post-hoc comparisons.

**Figure 5:**
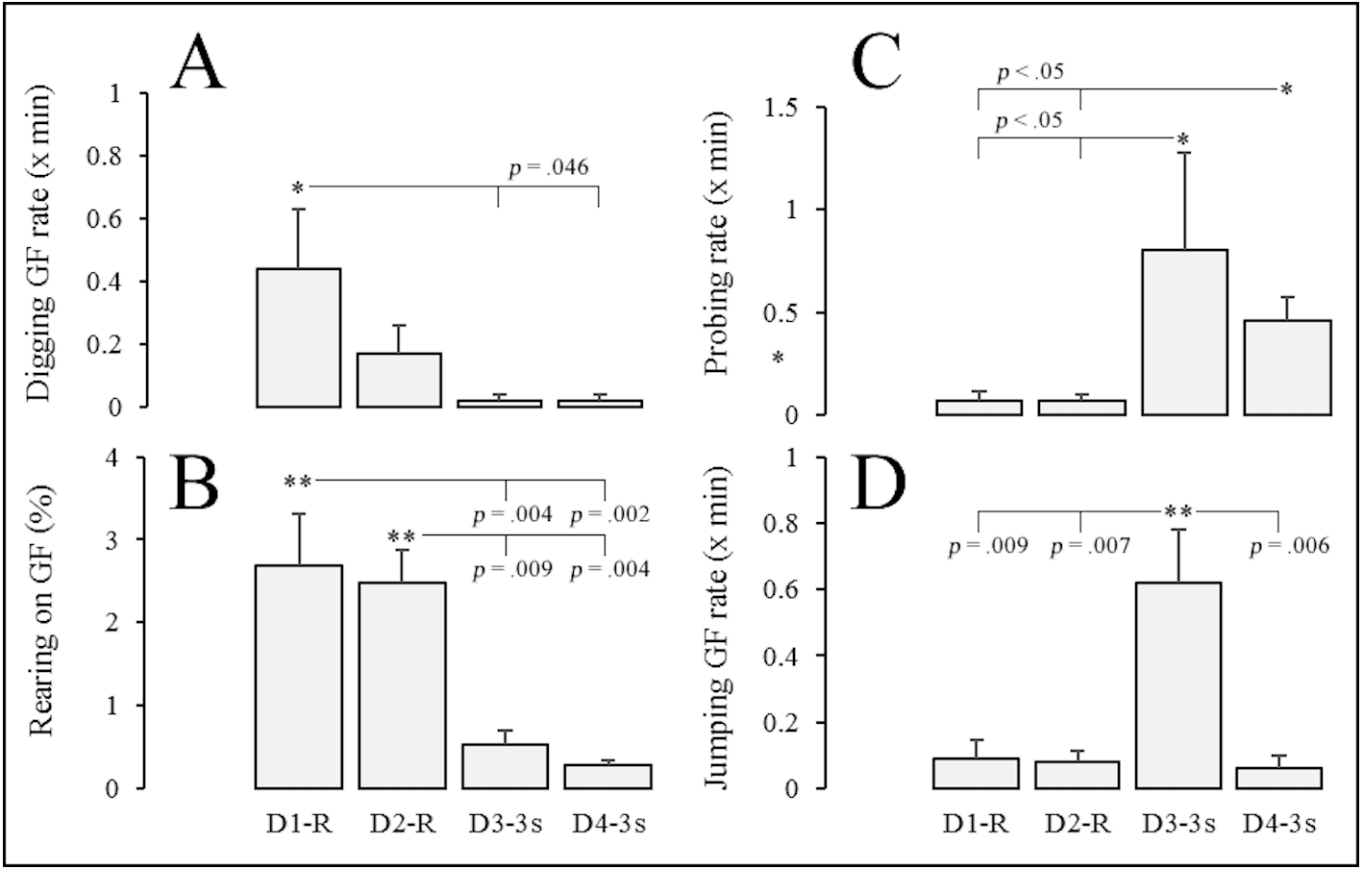
Exploratory behavior of gerbils across the different sessions of Experiment 2. The exploratory behavior of gerbils (*n* = 5) across the different sessions of Experiment 2–the digging rate on the grid floor (GF) is shown on (A), percentage of session time rearing on (B), rate of probing from the platform on (C), and jumping rate on (D). Error bars represent 1 S.E.M. Note: *D* = day of the experiment (session); *R* = foot shocks delivered on a random-time schedule of 30 s (RT-30s); *3 s*, = foot shocks delivered every 3 s. *, statistically significant post-hoc comparisons.

The results of Experiment 2 are summarized in Figs. 4 and 5. Repeated-measures ANOVA showed differences in the percentage of the session time allocated to the platform (*F*(3, 19) = 24.04, *p* < .001, ${\eta }_{p}^{2}=.86$) and the average duration of visits to the platform (*F*(3, 19) = 7.238, *p* = .005, ${\eta }_{p}^{2}=.65$) across sessions of the study (D1-R, D2-R, D3-3s, and D4-3s). As shown in Fig. 4A, post-hoc comparisons indicated that gerbils significantly spent more time on the platform during both sessions with periodic shocks (D3-3s and D4-3s) than during sessions in which the shocks were delivered randomly (D1-R and D2-R). By the last session of the experiment (D4-3s), the subjects were spending close to 90% of the session time on the platform. Cohen’s *d* calculations indicated large effects for the significant differences between D1-R–D3-3s (*p* = .004, *d* = 2.481), D1-R–D4-3s (*p* < .001, *d* = 3.604), D2-R–D3-3s (*p* = .002, *d* = 2.554), and D2-R–D4-3s (*p* < .001, *d* = 3.637).

As shown in Fig. 4C, the average visit to the platform was significantly longer during the second session of periodic shocks (D4-3s) compared to both sessions with shocks delivered with the random-time schedule (D1-R and D2-R). The average visit duration during that last session was 73 s. Cohen’s *d* calculations indicate large effects for the significant post-hoc comparisons D1-R –D4-3s (*p* = .008, *d* = 1.988) and D2-R–D4-3s (*p* = .01, *d* = 1.907).

No significant effect was observed for percentage of freezing time per session (*F*(3, 19) = 0.57, *p* = .644, ${\eta }_{p}^{2}=.13$) and step-down rates (*F*(3, 19) = 1.29, *p* = .321, ${\eta }_{p}^{2}=.27$) when comparing randomly delivered shocks (D1-R and D2-R) and periodic shocks (D3-3s and D4-3s)—See Figs. 4B and 4D.

The exploratory behavior of gerbils varied across sessions of the experiment (see Fig. 5). Repeated-measures ANOVA showed differences in three behaviors that were typically displayed on the grid floor: digging rate (*F*(3, 19) = 4.04, *p* = .034, ${\eta }_{p}^{2}=.53$), percentage of session time rearing (*F*(3, 19) = 13.33, *p* < .001, ${\eta }_{p}^{2}=.76$) and jumping rate (*F*(3, 19) = 8.39, *p* = .003, ${\eta }_{p}^{2}=.662$). In addition, the Friedman test of ranks showed differences in the rate of probing from the platform (*X*^2^(3) = 12.62, *p* = .006, *W* = .71).

As shown in Figs. 5A and 5B, post-hoc comparisons between random-time based and periodic delivery of foot shocks indicated a significant reduction on frequency of digging and percentage of time rearing. Cohen’s *d* calculations for these significant differences indicated large effects in digging (D1-R vs D3-3s, *p* = .046, *d* = 1.405; D1-R vs D4-3s, *p* = .046, *d* = 1.405) and rearing (D1-R vs. D4-3s, *p* = .002, *d* = 2.447; D1-R vs. D3-3s, *p* = .004, *d* = 2.165; D2-R vs. D3-3s, *p* = .009, *d* = 2.845; D2-R vs. D4-3s, *p* = .004, *d* = 3.382) across sessions.

Figure 5C shows that the rate of probing markedly increased when gerbils were switched from random to periodic foot shock delivery; Cohen’s *d* for significant differences in probing across these sessions indicated large effects (D1-R vs D3-3s, *p* < .05, *d* = 0.922; D1-R vs D4-3s, *p* < .05, *d* = 2.000; D2-R vs D3-3s, *p* < .05, *d* = 0.925; D2-R vs D4-3s, *p* < .05, *d* = 2.101). Finally, the rate of jumping on the grid floor (Fig. 5D) was significantly higher during the first day of periodic shocks (D3-3s) than all other sessions, with large effects across all comparisons (D3-3s vs. D1-R, *p* = .009, *d* = 1.952; D3-3s vs. D2-R, *p* = .007, *d* = 2.058; D3-3s vs. D4-3s, *p* = .006, *d* = 2.117).

### Discussion

Experiment 2 had a dual purpose: first, assessing a variation in the step-down protocol implemented during Experiment 1—effects of delivering the shocks on a RT 30 s schedule; and second, characterizing the activity of gerbils during the step-down protocol.

By the second session of the periodic shock delivery condition (D4-3s), the gerbils were spending nearly 90% of the session time on the platform, which replicated the findings of the 1.0 mA treatment conditions in Experiment 1 (see Fig. 2). The observation that such time allocations were significantly higher than those observed during sessions in which the shocks were delivered with a random schedule indicates that sporadic exposure to the foot shocks is not sufficient to produce substantial changes in preference for the platform area. This finding supports the effectiveness of the 1.0 mA protocol, and together with the observation that only two sessions with this intensity produced such significant changes, provides evidence for its suitability for further research.

Data on the average visit to the platform, together with the lack of differences in step-down rates across conditions, indicate that the effects of frequent shocks were not on the rate but on the length of the visits—i.e., the duration of each visit to the platform increased when gerbils were changed from random-time to periodic shock delivery. This finding is consistent with reports by Galvani, Riddel & Foster (1975) regarding differences between rats and gerbils in behavioral adjustments to step-down tasks. Galvani, Riddel & Foster (1975) found that in contrast to rats, which typically perform all their step-down responses after first exposure to the shocks, gerbils spaced a similar number of step-down responses over a longer period of observation.

The lack of differences in freezing time across the experimental conditions (percentages of session time remained approximately 35–40%) suggest that temporal patterning of shocks does not differentially affect this measure. Conversely, exploratory behavior did seem to be affected by a change from random-time to periodic shocks, as evidenced by the decrease in digging and rearing and the increments in probing and jumping. The observed patterning of these responses may relate to the notably-active defensive system of the gerbil (Ellard, 1993; Ellard, 1996; Ellard & Chapman, 1991), including its adjustment to the frequency and temporal distribution of aversive events. Variations in the probing response are of special relevance for further research (e.g., pharmacological) because they could not only be interpreted as a form of risk assessment but are also not displayed in step-down tasks by other popular rodent species (Galvani, Riddel & Foster, 1975).

## General Discussion

The aim of the present study was fourfold: (a) development of an extended step-down protocol that could systematically reproduce avoidance phenomena in gerbils using the VFC system; (b) use of this protocol to examine and compare the establishment, steady-state, extinction, and reacquisition of the step-down performance of gerbils with two foot-shock intensities (0.5 vs. 1.0 mA); (c) assessment of whether the effects of the first implemented protocol could also be obtained with shocks delivered with a random-time schedule (RT 30 s); and (d) characterization of the exploratory behavior of gerbils during the extended step-down task.

### Adaptation of the step-down protocol

The simple integration of a custom-made platform allowed for successful implementation of the designed step-down task for gerbils in the VFC system. This protocol produced reliable and clear evidence of avoidance learning in terms of rapid and major increments in freezing and on-platform time, as well as decrements in the rates of platform descents. The ease of adaptation of the protocol in a commercially available system, and the versatility of the system in terms of automatic scoring of activity and freezing measures, are expected to contribute to the need of a standard step-down protocol for this species. Further refinement and testing of this protocol could ultimately improve the commensurability of findings across laboratories, which in the past have implemented highly dissimilar protocols.

One major limitation of our protocol is that latency of the first step-down response—a widely used measure in related research (e.g., Borba Filho et al., 2015; Mina et al., 2014)—was not registered because no method for confining the subject to the platform prior to the start of the session was available. Further versions of the protocol should provide this datum, not only because it often has been the primary outcome of step-down tasks, but also because the relation between this measure and the others reported here could be interesting on its own. We are presently exchanging information with the developers of the VFC (MED Associates, Inc.) for this and related refinement purposes, including automated registering of the step-down and step-up responses.

### Effects of two foot-shock intensities (0.5 vs. 1.0 mA)

A comparison of the effects of the foot-shock intensities (0.5 versus 1.0 mA; Experiment 1) indicated that 1.0 mA shocks produced clearer and more reliable performance in terms of higher on-platform and freezing times, and more pronounced suppression of step-down responses. Reproduction of these effects during the analogue condition of Experiment 2 (D3–D4) confirmed the effectivity of that same intensity, and overall the suitability of our step-down protocol when implemented in the VFC. The extensive discrepancy in shock-intensity levels across the relevant literature (0.4–4.0 mA) warrants further research examining the effects of different intensities on the adjustment of gerbils to not only step-down tasks but also to other related protocols that have been implemented with diverse shock intensities (e.g., step-through type avoidance tasks; Imai et al., 2007; Ji et al., 2007; Kaundal & Sharma, 2011).

The observation that 1.0 mA foot shocks were efficient for producing clear and reliable avoidance behavior suggests that implementation of higher intensities is unnecessary for reproducing aversive conditioning effects in gerbils. Evidently, this notion requires further confirmation in studies manipulating more systematically different shock intensities and extend the analysis to female gerbils, which have been reported to perform less efficiently in step-down tasks (Galvani, Riddel & Foster, 1975). These efforts are needed to continue minimizing the pain and distress of laboratory animals through the refinement of experimental procedures, as strongly endorsed by agencies worldwide (National Research Council, 2011).

### Acquisition, extinction, and reacquisition of step-down performance

The gerbils’ steady step-down performance and its extinction and reacquisition were characterized in Experiment 1, which were efforts that have not been previously reported in the relevant literature. The on-platform time and freezing measures reached clear steady states in fewer sessions when the 1.0 mA foot shocks were scheduled. Psychological, pharmacological, and behavioral neuroscience research on emotion and learning phenomena involving operant conditioning (e.g., avoidance, punishment, or conditioned suppression; Aparicio, 2010; Knox, 2016) often requires the establishment of steady performance prior to testing the effects of other experimental manipulations (e.g., the effects of a given substance on performance). The step-down task reported herein offers an advantage over other experimental procedures (e.g., lever pressing in an operant chamber) such that steady performance could be obtained in a few short-duration sessions (6–8 sessions, 10 min each).

Although suppression of platform descents (step-down responses) was clearer and steadier across the 1.0 mA sessions, the rate of these responses often did not reach complete stability by the end of a given treatment condition, which could last up to 15 consecutive sessions. It is unclear why the gerbils did not completely stop descending from the platform and why the number of these responses continued to fluctuate after several sessions. However, Galvani, Riddel & Foster’s (1975) observation that the frequency of platform descents was higher in gerbils than in rats, warrants further research to extend these findings, which could result in a resumption of comparative efforts that were practically abandoned several decades ago. For example, it seems promising to explore patterns of step-down and exploratory responses (including probing, which Galvani, Riddel & Foster, 1975 only observed in gerbils) and analyze them in terms of the defensive behavior of these two species (e.g., unlike rats, which typically become almost permanently immobile, gerbils show facilitation of general activity while undergoing fear conditioning; Galvani, Riddel & Foster, 1975).

Similar to the case of acquisition, behavioral effects related to the extinction and reacquisition of the step-down task were clearer and steadier under 1.0 mA foot-shock conditions. Extinction conditions (i.e., foot-shock cessation) typically produced gradual decrements in freezing and on-platform time across sessions, whereas in the case of step-down responses, this procedure instead resulted in more sudden than gradual increments. Reacquisition of the task (i.e., performance during post-extinction treatment conditions) typically consisted of the rapid reestablishment of these three responses to levels observed during previous treatment conditions. Further studies could (a) explore differences in the pattern of immobility and on-platform time allocation versus platform descents during extinction conditions—i.e., changes in step-down responses were sudden, whereas variations in freezing and time allocation to the platform were typically gradual; and (b) examine the neurobiological and comparative aspects of the emotional-memory effects that were observed during the extinction and reacquisition conditions of the step-down protocol. In this regard, our step-down protocol shows promise for implementation in pharmacological and behavioral neuroscience research of complex emotional-memory phenomena (e.g., as an animal model of persistent fear memories characteristic of posttraumatic stress disorder—Perrine et al., 2016; Wurtz et al., 2016).

### Random-time versus response-dependent foot shocks

A comparison of the adjustment of gerbils to random-time versus response-contingent shocks indicated that random-time shocks did not produce substantial increments in time allocation to the platform that were characteristic of response-dependent shocks. Experiment 2’s data showed that changing from random-time to response-dependent shocks affected (a) the length of each visit to the platform, and not the rate of step-down responses per se, which remained at the same level during both conditions; and (b) the patterns of exploratory behavior of gerbils, in terms of a decrease in digging and rearing, and increments in probing and jumping.

Although no differences in freezing times were observed across conditions of Experiment 2, it is worth noting that the levels remained close to those observed during the 1.0 mA treatment conditions of Experiment 1 (approximately 40%; see Fig. 2). This outcome suggests that exposure to both types of shock delivery (random-time and response-dependent) increase freezing to levels above the characteristic of no-shock conditions (i.e., baseline), which during Experiment 1 typically remained at approximately 20%. Evidently, further research is needed to test this interpretation because no freezing baseline data were collected during Experiment 2.

Similarly, the low rates of step-down responses across both conditions in Experiment 2 resembled those of the subjects that were first exposed to 1.0 mA shocks during Experiment 1 (see S3 and S4 in Fig. 3), i.e., at approximately 1.5/min. This finding, and the lack of differences across conditions in Experiment 2, suggested that both types of shock delivery suppressed step-down responses to levels below no-shock conditions (baselines), which during Experiment 1 typically remained close to 3 per min (see Fig. 3). Again, this interpretation requires further systematic testing because no baseline data were collected during Experiment 2.

### Exploratory and defensive behavior

The exploratory behavior of gerbils that accompanied traditionally measured adjustment to the step-down task—freezing and on-platform time and frequency of platform descents—consisted of the suppression of digging and rearing responses, and increments in probing and jumping behavior. The patterning of freezing, platform-descents, and exploratory behavior may be related to the active defensive system of the gerbil (Ellard, 1993; Ellard, 1996; Ellard & Chapman, 1991), including its adjustment to the frequency and temporal distribution of aversive events. Further detailed research examining the patterning of probing behavior seems particularly promising considering its potential for comparative research, since it has not been reported in other rodent models and could possibly be interpreted as a form of risk assessment.

## Conclusions

The first experiment addressed the discrepancy in foot-shock intensities reported in previous research with gerbils exposed to step-down procedures, and the lack of information on this model’s long-term performance, extinction, and behavioral patterning during these tasks. The extended protocol designed to that end showed that 1.0 mA shocks produced reliable and clear evidence of avoidance learning, extinction, and reacquisition across different behavioral measures (freezing, on-platform time, and step-down responses). These findings demonstrate the feasibility of our protocol to study this type of learning and emotional phenomena in gerbils, and suggest that using shocks of higher intensities is unnecessary for reproducing reliable aversive-conditioning effects in this animal model. This protocol shows promise for implementation in pharmacological and behavioral neuroscience research of complex emotional-memory phenomena, and for further refinement that could improve the commensurability of findings across laboratories. The second experiment demonstrated that an alternate protocol consisting of random delivery of shocks does not result in the acquisition of avoidance behavior that was observed in the first experiment. It also allowed to characterize gerbils’ exploratory behavior during the step-down task, which consisted of suppression of digging and rearing responses, and increments in probing and jumping behavior. The wide range of responses displayed by gerbils during aversive procedures—perhaps beyond that observed in mice or rats—calls for resuming comparative efforts (e.g., Pérez-Acosta et al., 2016;  Wang et al., 2017), and suggests its better potential for translational clinical studies. Of special interest is the probing behavior, which could be interpreted as risk assessment and has not been reported in other rodent species exposed to step-down and similar tasks.

##  Supplemental Information

10.7717/peerj.4009/supp-1 Appendix S1 Snapshot of a video recording obtained with the Video Fear Conditioning System (VFC) Snapshot of a video recording (original at 640 × 480 px) showing the interior of the experimental chamber with near-infrared light.

10.7717/peerj.4009/supp-2 Appendix S2 VFC and JWatcher files VFC files for running experimental protocols (including the Bancroft & Bourret’s 2008 EXCEL macro for creating the random time 30 s values). Jwatcher 1.0 files for scoring of the videos (focal master file) and analyzing the output (focal analysis file).

10.7717/peerj.4009/supp-3 Appendix S3 Sequence of the treatment conditions during Experiment 1 Sequence of the treatment conditions to which each subject was exposed during Experiment 1. Note: BL, baseline condition; 0.5 mA, foot shocks of 0.5 mA; 1.0 mA, foot shocks of 1.0 mA.

10.7717/peerj.4009/supp-4 Appendix S4 Definitions of exploratory behavior Definitions of exploratory behaviors scored during Experiment 2 based on Hurtado-Parrado & Lopez-Lopez (2015).

## Additional Information and Declarations

### Data Availability

The following information was supplied regarding data availability: Hurtado-Parrado, Camilo. 2017. Manuscript Mongolian Gerbil Behavior during a Step-down Task. Open Science Framework. September 23. DOI: 10.17605/OSF.IO/FN4XS.
