# Supplementary figures and images for: Assessing Mongolian gerbil emotional behavior: effects of two shock intensities and response-independent shocks during an extended inhibitory-avoidance task
Source: PeerJ. 2017 Nov 13;5:e4009. doi: 10.7717/peerj.4009 (PMC5689020; doi:10.7717/peerj.4009)

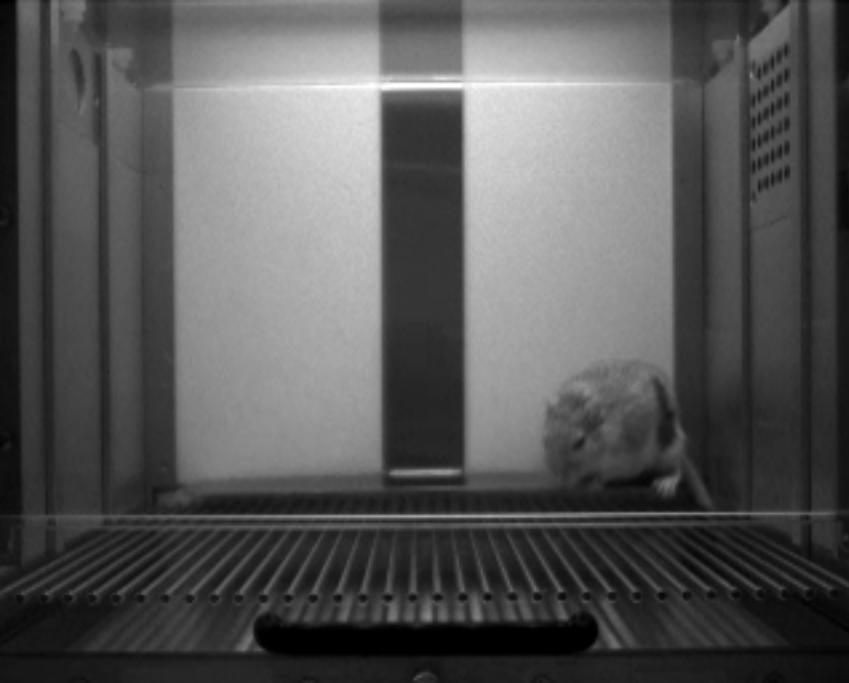

Supplement: Appendix S1 — Snapshot of a video recording (original at 640 × 480 px) showing the interior of the experimental chamber with near-infrared light. [file peerj-05-4009-s001.pdf]
