# Supplementary material for: Assessing Mongolian gerbil emotional behavior: effects of two shock intensities and response-independent shocks during an extended inhibitory-avoidance task
Source: PeerJ. 2017 Nov 13;5:e4009. doi: 10.7717/peerj.4009 (PMC5689020; doi:10.7717/peerj.4009)
Supplement: Appendix S2 — VFC files for running experimental protocols (including the Bancroft & Bourret’s 2008 EXCEL macro for creating the random time 30 s values). Jwatcher 1.0 files for scoring of the videos (focal master file) and analyzing the output (focal analysis file). [file peerj-05-4009-s002.zip › Protocols sharing/VFC/Bancroft, Bourret - 2008 - Generating variable and random schedules of reinforcement using Microsoft Excel macros.pdf]

JOURNAL OF APPLIED BEHAVIOR ANALYSIS 2008, **41,** 227–235 NUMBER 2 (SUMMER 2008)

GENERATING VARIABLE AND RANDOM SCHEDULES OF REINFORCEMENT USING MICROSOFT EXCEL MACROS

STACIE L. BANCROFT AND JASON C. BOURRET

NEW ENGLAND CENTER FOR CHILDREN

Variable reinforcement schedules are used to arrange the availability of reinforcement following varying response ratios or intervals of time. Random reinforcement schedules are subtypes of variable reinforcement schedules that can be used to arrange the availability of reinforcement at a constant probability across number of responses or time. Generating schedule values for variable and random reinforcement schedules can be difficult. The present article describes the steps necessary to write macros in Microsoft Excel that will generate variable-ratio, variable-interval, variable-time, random-ratio, random-interval, and random-time reinforcement schedule values.

DESCRIPTORS: random interval, random ratio, random time, schedule generation, schedules of reinforcement, variable interval, variable ratio, variable time

---

Variable schedules of reinforcement minimize problems associated with the delivery of reinforcement according to fixed schedule parameters. In fixed-ratio (FR), fixed-interval (FI), or fixed-time (FT) schedules, reinforcement is more probable after the passage of a given amount of time or following a given number of responses, and the types of temporal response patterning observed under FR and FI schedules are less likely to occur under variable-ratio (VR) and variable-interval (VI) schedules (Catania, 2006).

In VR schedules, reinforcement follows a varying number of responses with a specified mean value (Catania, 2006). Applied behavior analysts have used VR schedules in the examination of reinforcement effects on a range of topographies of behavior, including compliance (Neef, Shafer, Egel, Cataldo, & Parrish, 1983), choice of seated activity or food in obese and nonobese women (Saelens & Epstein, 1996), improving quality of handwriting among children (Veena, Romate, & Bhogle, 2002), and correct responding on rhyming tasks used to assess early literacy (Broussard, VanDerHeyden, Fabre, Stanley, & Ordoynne, 2006). These schedules have also been used to establish steady-state responding as a baseline for examination of the effects of experimental manipulations (Carr, Kellum, & Chong, 2001; Wylie & Grossmann, 1988).

In VI schedules, the first response following varying intervals of time with a specified mean value is reinforced (Catania, 2006). These schedules have been used to examine the effects of social reinforcement on academic engagement (Martens, Lochner, & Kelly, 1992), reinforcement rate and reinforcer quality effects on time allocation to math problems (Neef, Mace, Shea, & Shade, 1992), and reinforcement-schedule changes given different types of instruction (Bicard & Neef, 2002; Neef et al., 2004).

Variable-time (VT) schedules arrange reinforcement delivery following intervals of time with an overall mean value, with no response contingency in place. These schedules have been used in a number of studies. Saudargas, Madsen, and Scott (1977) showed an increase in student academic performance when progress reports were sent home on a VT schedule compared to an FT schedule. Van Camp, Lerman, Kelley, Contrucci, and Vorndran (2000) found the use of FT and VT schedules to be equally effective in decreasing problem behavior. Carr et al. (2001) compared the use of

---

Address correspondence to Jason Bourret, New England Center for Children, 33 Turnpike Road, Southborough, Massachusetts 01772 (e-mail: jbourret@necc.org).

doi: 10.1901/jaba.2008.41-227

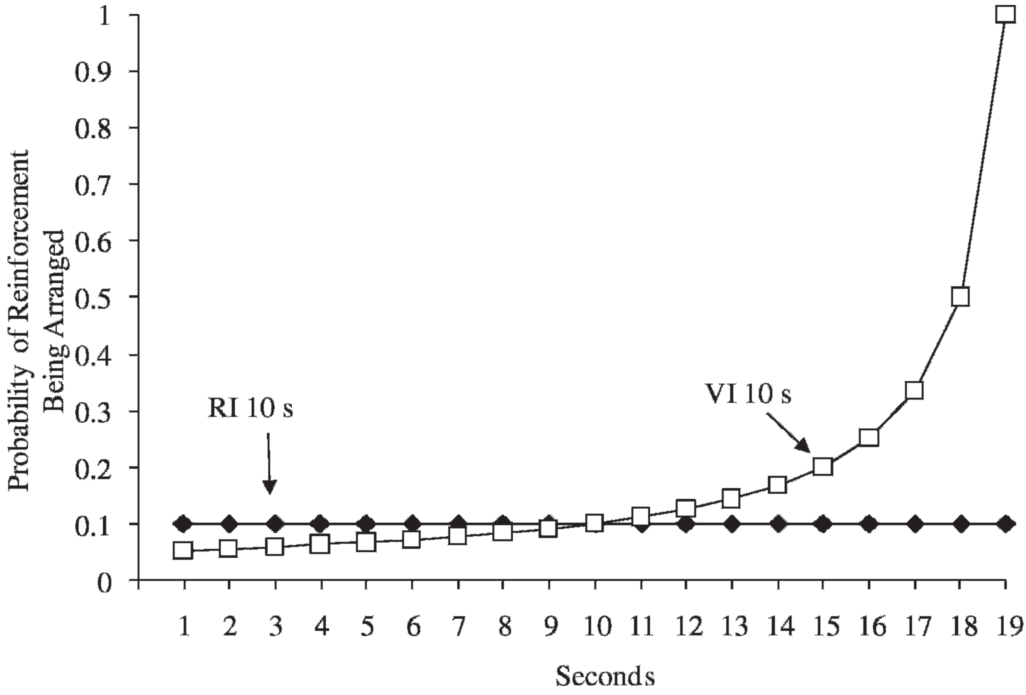

Figure 1. The probability of reinforcement being arranged for a VI 10-s schedule with a rectangular distribution of component values and an RI 10-s schedule.

FT and VT schedules in decreasing behavior previously maintained on a VR schedule of reinforcement and found both to be effective.

Some types of variable schedules, however, are limited in that the probability of reinforcement being scheduled or delivered increases across time or number of responses. For example, Figure 1 shows the probability of reinforcement being arranged in a VI 10-s schedule in which the component values range from 1 s to 19 s, with a rectangular distribution (i.e., all values within the array occur with equal frequency). Reinforcement has a 1 in 19 chance of being arranged at Second 1. If reinforcement is not arranged, there is a 1 in 18 chance of being arranged at Second 2, a 1 in 17 chance at Second 3, a 1 in 16 chance at Second 4, and so on. The probability of reinforcement being arranged increases as time passes, and some level of temporal patterning may emerge (Catania & Reynolds, 1968). Even though other types of variable schedules have been developed that limit temporal patterning of responding by arranging particular distributions of reinforcement in time (e.g., Fleshler & Hoffman, 1962; Millenson, 1966), random schedules avoid generating temporal patterning by keeping the probability of reinforcement constant over time. For example, in a random-interval (RI) 10-s schedule, at every second there is a .1 probability of reinforcement being arranged (see Figure 1). Once reinforcement has been arranged, the next response produces reinforcement. In the RI 10-s schedule, reinforcement has a 1 in 10 chance of being arranged at Second 1, a 1 in 10 chance of being arranged at Second 2, a 1 in 10 chance of being arranged at Second 3, and so on. Even though random schedules can be useful in generating relatively constant probabilities of responding over time, they are limited in that, because the values in the array are random, the clinician or researcher cannot directly control the values in the distribution. Thus, random schedules could

potentially lead to undesirable patterns of responding if, for example, several small values or several large values happen to be selected in sequence.

Researchers and clinicians may find variable and random schedules useful for a variety of purposes. For example, in thinning the schedule of noncontingent reinforcement delivery, reinforcement may initially be present continually and then changed to successively leaner random-time (RT) schedules (e.g., RT 5 s, RT 10 s, RT 30 s, RT 60 s, RT 120 s, RT 300 s). Similarly, a random-ratio (RR) schedule may be useful in fading response requirements from an FR 1 schedule to a less dense VR schedule. Variable and random schedules also may be used in production of higher rates of responding than those observed under continuous reinforcement or in generating responding that is maintained longer under extinction. Variable schedules may be useful if there is a concern that the amount of reinforcement being delivered might function as an abolishing operation. Intermittent reinforcement can be used to maintain behavior with a lower density of reinforcement per unit time. Another use for these schedules may be in establishing baseline responding with a relatively constant rate over time in order to evaluate the effects of manipulations of the independent variable.

Methods of generating variable or random schedules that are not automated may be time consuming and complicated compared to computerized methods of schedule generation. Below, we provide methods for writing Microsoft Excel macros that can generate a large number of schedule values rapidly for VR and RR, VI and RI, and VT and RT schedules.

METHOD

Writing the Macros

Variable and random schedules can be generated using a Microsoft Excel macro. To create a macro, first open Excel. Under the Tools menu, click on Macro or on the keyboard press Alt F8. The Macro window will open. In the space that says "Macro name:" type the name of the macro you will be writing (e.g., VR) and click on "Create." The macro can be written now. The following are step-by-step instructions for developing macros that generate VR, VI, VT, RR, RI, and RT schedules. In addition, the steps required to use the macros once they have been created also are included.

Variable-ratio schedules. Figure 2 shows the components of a macro that will produce a VR schedule of reinforcement. This macro generates a list of numbers that can be read as response requirements for reinforcement delivery. Any VR schedule can be generated using the code in Figure 2 by making simple modifications to the worksheet. In Line A, "Sub" should be followed by the name of the macro you previously entered in the macro window. Because we are creating a VR schedule, the line reads "Sub VR ()." Open and closed parentheses follow the schedule name. The code in Line B declares the array variable "arrayvalue" and gives it 100 spaces to store values. These spaces will be used to store the values of each ratio composing the VR distribution. Line C clears all previous items in the first column of the spreadsheet for the first 600 cells. This is where the schedule values will be listed.

In Lines D and E, the variable "valuenum" is set to 0 and "rownum" to 1. These variables will be used later in the code. Lines F through I include a "do … until" statement, which allows the code to repeat until some condition in the statement is true. These lines of code are responsible for storing the user-entered values that will make up the VR distribution. Line F begins this statement with "do." In Line G, 1 is added to "valuenum." In Line H, the value in the array is set to the distribution value listed in Column B of the worksheet. This loop continues until all values of the VR distribution have been entered into the array "arraynum." A second "do … until" loop is shown in Lines J through N. These lines of code are responsible

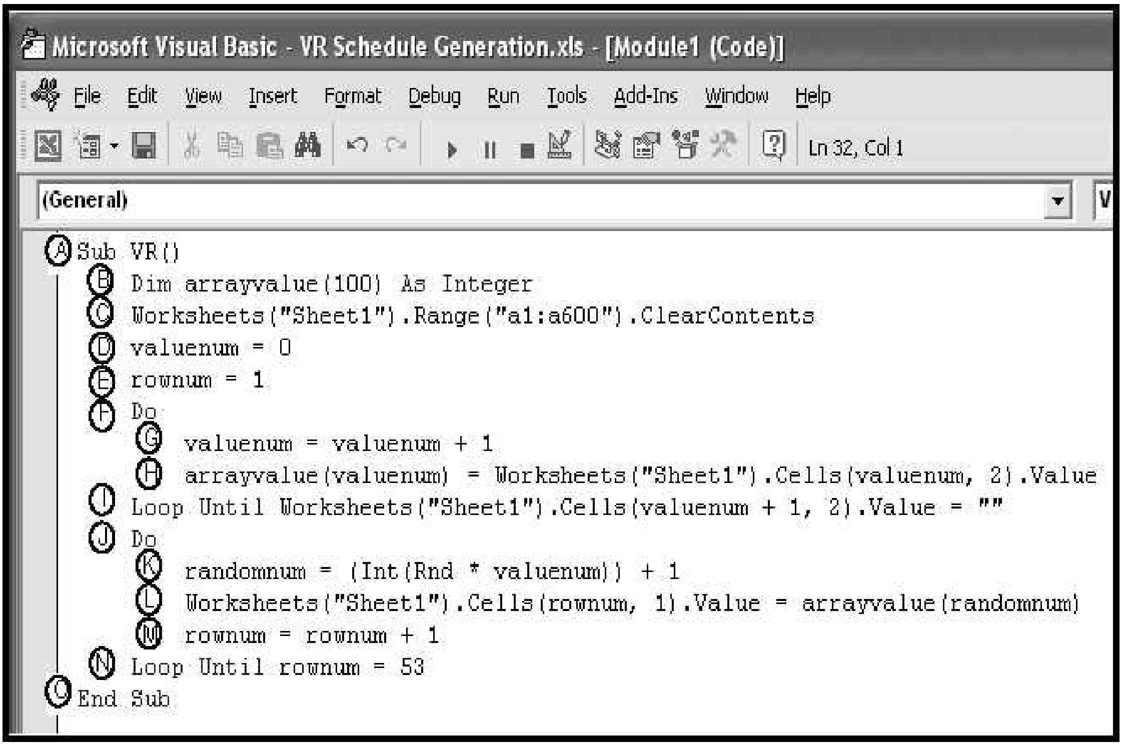

Figure 2. An Excel macro for VR schedule generation as it would look in the edit window of Excel Visual Basic. The circled letters are not part of the code; they are used to reference lines in the code described in the text.

for generating and displaying the schedule of reinforcement. In Line J, the statement begins with "Do." In Lines K and L, one of the members of the VR distribution is selected randomly and entered into a cell in Column A. In Line M, a variable is incremented so that the next VR value will be placed in the next cell of Column A. In Line N, the loop ends just prior to the 53rd value being selected. Each running of the macro will write 52 VR values into Column A of the worksheet (this is the number of rows that will be printed on one page without changing the cell height). Line O ends the macro.

Variable-interval schedules. Figure 3 shows the macro for VI schedule generation. In Line A, "sub" is followed by the macro name "VI" and open and closed parentheses. Lines B through H declare the variables to be used in the macro. Lines I through N include the first "do … until" statement, which is identical to the first loop in the VR macro. Lines O through W include the second "do … until" statement. This loop is also similar to the second loop in the VR, with the exceptions that the macro will generate a distribution of values for a user-entered session duration and, thus, the number of values generated is not set at 52. For example, for a VI 30-s schedule during a 2-min session, a relatively small number of values would be obtained prior to the end of the session, but for a 3-hr session, a relatively large number of values would be generated. Further, the values composing the schedule are written into the worksheet in a cumulative minutes-and-seconds format (i.e., the real time in the session at which reinforcement should be arranged is shown). The code used for the VI macro also can be used for VT schedule generation, the only difference being the manner in which the therapist conducts the schedule.

```
(A) Sub VI()
  (B) Dim arrayvalue(100) As Integer
  (C) Worksheets("Sheet1").Range("a1:a600").ClearContents
  (D) entry = 1
  (E) final = 1
  (F) valuenum = 0
  (G) rownum = 1
  (H) totaltime = 0
  (I) Do
    (J) valuenum = valuenum + 1
    (K) entry = Worksheets("Sheet1").Cells(valuenum, 2).Value
    (L) final = Worksheets("Sheet1").Cells(valuenum + 1, 2).Value
    (M) arrayvalue(valuenum) = entry
  (N) Loop Until final = ""
  (O) Do
    (P) randomnum = (Int(Rnd * valuenum)) + 1
    (Q) totaltime = totaltime + arrayvalue(randomnum)
    (R) If totaltime <= Worksheets("Sheet1").Cells(1, 3).Value Then
      (S) timeplace = TimeSerial(0, totaltime, 0)
      (T) Worksheets("Sheet1").Cells(rownum, 1).Value = FormatDateTime(timeplace, vbShortTime)
      (U) rownum = rownum + 1
    (V) End If
  (W) Loop Until totaltime >= Worksheets("Sheet1").Cells(1, 3).Value
(X) End Sub
```

Figure 3. Code for an Excel macro for VI or VT schedule generation. The circled letters are not part of the code; they are used to reference lines in the code described in the text.

Random-ratio schedules. The macro for RR schedule generation is shown in Figure 4. The macro name "RR" is in Line A and is followed by open and closed parentheses. Line B clears the cells in Column A of the worksheet, which is where the values of the RR schedule will be listed. In Line C, the variable name "rownum" is set equal to 1. Line D begins a loop with the "Do" statement. In Lines E and F, the "responsenum" variable is set to 1, and the "randomnum" variable is set to a random number between 0 and 1. In Line G, the mean ratio value is retrieved from the worksheet and converted into the probability of each response producing reinforcement. For example, in an RR 5 schedule there is a .2 probability of reinforcement being arranged after each response. If the probability generated is not .2 or below, the response being checked is increased by 1 in Line H, and a new random number is generated in Line I. In Line J, the macro loops back to Line G. This loop continues until the probability generated is less than or equal to .2, and the response that will produce reinforcement is identified. That is, if reinforcement is not arranged for Response 1, Response 2 is checked, then Response 3, and so on. This response number is then written into the spreadsheet (Line K). Lines L and M set the number of values generated such that 52 are listed in the worksheet (the number that fit on one page of a standard Excel spreadsheet). In

```
Ⓐ Sub RR()
  Ⓑ Worksheets("Sheet1").Range("a1:a600").ClearContents
  Ⓒ rownum = 1
  Ⓓ Do
      Ⓔ responsenum = 1
      Ⓕ randomnum = Rnd
      Ⓖ Do While randomnum > (1 / Worksheets("Sheet1").Cells(1, 2).Value)
          Ⓗ responsenum = responsenum + 1
          Ⓘ randomnum = Rnd
      Ⓙ Loop
      Ⓚ Worksheets("Sheet1").Cells(rownum, 1).Value = responsenum
      Ⓛ rownum = rownum + 1
  Ⓜ Loop Until rownum = 53
Ⓝ EndSub
```

Figure 4. Code for an Excel macro for RR schedule generation. The circled letters are not part of the code; they are used to reference lines in the code described in the text.

Line N, the macro is ended. This macro is sufficient for generating RR schedules with a variety of different mean values. The average ratio is determined by user input, which will be discussed in the section on running the macro.

Random-interval schedules. The macro for RI schedule generation works in a similar manner to the RR macro. Rather than determining the probability of reinforcement for each response, the RI macro generates a probability for every second, and the times at which reinforcement should be arranged are entered into the worksheet. Figure 5 shows the components of a macro that will generate an RI schedule of reinforcement. In Line A, as in the RR schedule, the "RI" indicates the name of the macro. The code in Line B clears all previous items in the first column for the first 600 cells. In Line C the variable "rownum" is set to zero, and in Line D the counter is set to a range of 1 to whatever value is entered into Column C, Row 1 of the macro. For example, to generate intervals for a 5-min (300-s) session, 300 would be entered into the Column C, Row 1 cell of the worksheet once the macro has been created. The counter variable will start at 1 and proceed through each of the possible seconds for which reinforcement could be arranged (in this example, 300). In Line E, "randomnum" is set as a random number between 0 and 1. In Line F, the mean interval value is retrieved from the worksheet and converted into the probability of reinforcement being arranged for each second. For example, in an RI 10-s schedule there is a .1 probability of reinforcement arranged for each second. When the random number is less than or equal to the probability of reinforcement being arranged for a given second, that second of the session is written into Column A of the worksheet (Lines G, H, and I). For instance, if there is a .1 probability of reinforcement being arranged at Second 5, and the random number generates a number less than or equal to .1, Second 5 is entered into the worksheet. If the random number generated is larger than .1, Second 5 is not entered into the worksheet, and a random number is then generated to determine if reinforcement should

```
Ⓐ Sub RI()
  Ⓑ Worksheets("Sheet1").Range("a1:a600").ClearContents
  Ⓒ rownum = 0
  Ⓓ For counter = 1 To Worksheets("Sheet1").Cells(1, 3).Value
   Ⓔ  randomnum = Rnd
     Ⓕ If randomnum <= (1 / Worksheets("Sheet1").Cells(1, 2).Value) Then
       Ⓖ rownum = rownum + 1
       Ⓗ timeplace = TimeSerial(0, counter, 0)
       Ⓘ Worksheets("Sheet1").Cells(rownum, 1).Value =   FormatDateTime(timeplace, vbShortTime)
     Ⓙ End If
  Ⓚ Next counter
Ⓛ End Sub
```

Figure 5. Code for an Excel macro for RI or RT schedule generation. The circled letters are not part of the code; they are used to reference lines in the code described in the text.

be arranged for Second 6. Lines H and I convert the value into a cumulative minutes-and-seconds format and place it into the first column of the worksheet. Line J is the end of the "if … then" statement. The process repeats until the counter has gone through each second of the session, and all seconds at which reinforcement should be arranged have been written into Column A in the worksheet. The RI macro also can be used for RT schedule generation, the only difference being the way in which the schedule is implemented. For example, in an RT 20-s schedule (i.e., a .05 probability of reinforcement being arranged at each second) the therapist may deliver a reinforcer at each second of the session indicated in Column A, regardless of responding.

Running the Macros

To access the macro after it has been created and saved, open the saved Excel file. The user should then enter the desired schedule values into the worksheet prior to running the macro. For the VR macro, enter the entire distribution of values in Column B (e.g., for a VR 5 with a rectangular distribution and a range of values from 1 to 9, the numbers 1, 2, 3, 4, 5, 6, 7, 8, and 9 should be typed into Column B, beginning with Row 1). When the VR macro is run, these will be the values that are used to generate the schedule.

For the VI or VT macros, enter the distribution of values in seconds in Column B and the total number of seconds in the session in the first row of Column C. For example, for a VI 30-s schedule conducted over a 10-min session, the values entered into Column B might be 15, 22, 30, 38, and 45, and the value entered into Column C, Row 1 would be 600. The VI macro will use the values in Column B as the members of the VI distribution and the value entered into Column C, Row 1 as the time (in seconds) at which the session ends. When the macro is run, the successive intervals composing the schedules will be written cumulatively into Column A. For example, Column A might read :15, :53, 1:23, 2:08, and so on. If the macro is being used to generate values for a VI schedule, two separate timers should be used during a session. The first should stop when reinforcement is scheduled to be arranged, the next response should be reinforced, and the schedule timer should be restarted. We recommend the use of a separate

timer to measure total session duration in this case. If the macro is being used to generate values for a VT schedule, only one timer is necessary, and reinforcement should be delivered at each second indicated by the schedule.

For RR schedule generation, enter the desired mean ratio value in Column B, Row 1 of the worksheet. For example, for an RR 5 schedule, 5 should be entered into the Column B, Row 1 cell of the worksheet. For the RI and RT macros, enter the desired mean interval in seconds into the first cell of Column B in the worksheet. The duration of the session in seconds should be entered into the first row of Column C. For example, if generating an RI 10-s schedule to be used in a 5-min (300-s) session, enter 10 into the first row of Column B and enter 300 into the first row of Column C. Once the RI or RT schedules have been generated, they are conducted in a manner identical to the VI or VT schedules, respectively.

We recommend using different Excel files for each schedule type to avoid any potential formatting problems. The formats used by the macros in writing the schedule values into the worksheet differ between ratio and interval or time schedules (i.e., for ratio schedules a number is generated, for interval schedules a time is generated). By using separate files for VR, VI, RR, and RI schedules, any formatting conflicts can be avoided. To run any of the macros once the appropriate Excel file is open, press alt F8, highlight the schedule to be run, and click on run. This procedure is then repeated to generate additional schedules.

DISCUSSION

The macros described here were written and tested on Excel 2003 for Windows 2000. These functions should also operate on all previous versions of Excel and Windows operating systems. Although there are other methods available for creating variable and random schedules of reinforcement, the instructions above should be helpful in generating values for these schedules quickly and easily with no need for additional software beyond Excel.

REFERENCES

Bicard, D. F., & Neef, N. A. (2002). Effects of strategic versus tactical instructions on adaptation to changing contingencies in children with ADHD. *Journal of Applied Behavior Analysis*, *35*, 375–389.

Broussard, C., VanDerHeyden, A., Fabre, M., Stanley, J., & Ordoynne, S. (2006). The effect of performance contingencies on correct responding on measures of early literacy. *Journal of Evidence-Based Practices for Schools*, *7*, 94–113.

Carr, J. E., Kellum, K. K., & Chong, I. M. (2001). The reductive effects of noncontingent reinforcement: Fixed-time versus variable-time schedules. *Journal of Applied Behavior Analysis*, *34*, 505–509.

Catania, A. C. (2006). *Learning* (interim [4th] ed.). Cornwall-on-Hudson, NY: Sloan.

Catania, A. C., & Reynolds, G. S. (1968). A quantitative analysis of the responding maintained by interval schedules of reinforcement. *Journal of the Experimental Analysis of Behavior*, *11*, 327–383.

Fleshler, M., & Hoffman, H. S. (1962). A progression for generating variable-interval schedules. *Journal of the Experimental Analysis of Behavior*, *5*, 529–530.

Martens, B. K., Lochner, D. G., & Kelly, S. Q. (1992). The effects of variable-interval reinforcement on academic engagement: A demonstration of matching theory. *Journal of Applied Behavior Analysis*, *25*, 143–151.

Millenson, J. R. (1966). Probability of response and probability of reinforcement in a response defined analogue of an interval schedule. *Journal of the Experimental Analysis of Behavior*, *9*, 87–94.

Neef, N. A., Mace, F. C., Shea, M. C., & Shade, D. (1992). Effects of reinforcer rate and reinforcer quality on time allocation: Extensions of matching theory to educational settings. *Journal of Applied Behavior Analysis*, *25*, 691–699.

Neef, N. A., Marckel, J., Ferreri, S., Jung, S., Nist, L., & Armstrong, N. (2004). Effects of modeling versus instructions on sensitivity to reinforcement schedules. *Journal of Applied Behavior Analysis*, *37*, 267–281.

Neef, N. A., Shafer, M. S., Egel, A. L., Cataldo, M. F., & Parrish, J. M. (1983). The class specific effects of compliance training with do and don't requests: Analogue analysis and classroom application. *Journal of Applied Behavior Analysis*, *16*, 81–99.

Saelens, B. E., & Epstein, L. H. (1996). Reinforcing value of food in obese and non-obese women. *Appetite*, *27*, 41–50.

Saudargas, R. W., Madsen, C. H., Jr., & Scott, J. W. (1977). Differential effects of fixed- and variable-time feedback on production rates of elementary school children. *Journal of Applied Behavior Analysis*, *10*, 673–678.

Van Camp, C. M., Lerman, D. C., Kelley, M. E., Contrucci, S. A., & Vorndran, C. M. (2000). Variable-time reinforcement schedules in the treatment of socially maintained problem behavior. *Journal of Applied Behavior Analysis*, *33*, 545–557.

Veena, A. S., Romate, J., & Bhogle, S. (2002). Improving children's handwriting: An operant model approach. *Journal of the Indian Academy of Applied Psychology*, *28*, 25–30.

Wylie, A. M., & Grossmann, J. A. (1988). Response reduction through the superimposition of continuous reinforcement: A systematic replication. *Journal of Applied Behavior Analysis*, *21*, 201–206.

Received February 21, 2007
Final acceptance April 24, 2007
Action Editor, James Carr
