# Supplementary material for: Assessing Mongolian gerbil emotional behavior: effects of two shock intensities and response-independent shocks during an extended inhibitory-avoidance task
Source: PeerJ. 2017 Nov 13;5:e4009. doi: 10.7717/peerj.4009 (PMC5689020; doi:10.7717/peerj.4009)
Supplement: Appendix S3 — Sequence of the treatment conditions to which each subject was exposed during Experiment 1. Note: BL, baseline condition; 0.5 mA, foot shocks of 0.5 mA; 1.0 mA, foot shocks of 1.0 mA. [file peerj-05-4009-s003.docx]

**Appendix 3**

*Sequence of the treatment conditions to which each subject was exposed during Experiment 1.*

| Subject | Conditions | | | | | | | |
| --- | --- | --- | --- | --- | --- | --- | --- | --- |
| S1 and S2 | BL | 0.5-mA | BL | 0.5 mA | BL | 1.0-mA | BL | 1.0-mA |
| S3 and S4 | BL | 1.0-mA | BL | 1.0-mA | BL | 0.5 mA | BL | 0.5 mA |

Note: *BL* = baseline condition; *0.5 mA* = foot shocks of 0.5 mA; *1.0 mA* = foot shocks of 1.0 mA.
