# Supplementary material for: Assessing Mongolian gerbil emotional behavior: effects of two shock intensities and response-independent shocks during an extended inhibitory-avoidance task
Source: PeerJ. 2017 Nov 13;5:e4009. doi: 10.7717/peerj.4009 (PMC5689020; doi:10.7717/peerj.4009)
Supplement: Appendix S4 — Definitions of exploratory behaviors scored during Experiment 2 based on Hurtado-Parrado & Lopez-Lopez (2015). [file peerj-05-4009-s004.docx]

**Appendix 4.**

*Definitions of exploratory behaviors scored during Experiment 2 based on Hurtado-Parrado et al. (2015)*

| Behavior | Definition |
| --- | --- |
| Probing (from the platform) | While on the platform - i.e., with four legs away from the grid floor - the gerbil quickly extended one or both forepaws, touched the grid floor, and returned to the original posture. |
| Digging (on the grid floor) | The gerbil performed rapid back and forth movements with its front paws, which alternated with movements of its hind legs that were directed toward the grid floor, walls, or corners. These movements were observed while the animal was hunched or standing upright (rear) against the walls of the experimental chamber. |
| Rearing (on the grid floor) | The gerbil stood upright on its hind legs with a straight back, and both of its front paws were off the floor and may or may not have been in contact with the walls of the experimental chamber. |
| Jumping (on the grid floor) | The gerbil pushed itself off the ground with its hind legs in a ‘‘jump-like’’ vertical movement. |
